# Common and unique features of viral RNA-dependent polymerases

**DOI:** 10.1007/s00018-014-1695-z

**Published:** 2014-08-01

**Authors:** Aartjan J. W. te Velthuis

**Affiliations:** 1 Molecular Virology Laboratory, Department of Medical Microbiology, Center of Infectious Diseases, Leiden University Medical Center, PO Box 9600, 2300 RC Leiden, The Netherlands; 2 Sir William Dunn School of Pathology, University of Oxford, South Parks Road, Oxford, OX1 3RE UK

**Keywords:** RNA virus, Retrovirus, RdRp, Reverse transcriptase, Dynamics

## Abstract

Eukaryotes and bacteria can be infected with a wide variety of RNA viruses. On average, these pathogens share little sequence similarity and use different replication and transcription strategies. Nevertheless, the members of nearly all RNA virus families depend on the activity of a virally encoded RNA-dependent polymerase for the condensation of nucleotide triphosphates. This review provides an overview of our current understanding of the viral RNA-dependent polymerase structure and the biochemistry and biophysics that is involved in replicating and transcribing the genetic material of RNA viruses.

## Introduction

Infections with RNA viruses place a constant burden on our healthcare systems and economy [1, 2]. Over the past century, this has been particularly true for infections with the *Human immunodeficiency virus* *1* (HIV-1), *Influenza A virus* (IAV), *Rotavirus* (RotaV), *West Nile virus* (WNV), *Dengue virus* (DV), *Measles virus* (MV), and the *Porcine reproductive and respiratory syndrome* *virus* (PRRSV) [2–6]. But also emergent RNA viruses can have considerable consequences, such as the *Severe acute respiratory syndrome*-*related coronavirus* (SARS-CoV) in 2003 [7] and, more recently, the *Middle East Respiratory Syndrome coronavirus* (MERS-CoV) [8] and the *Schmallenberg virus* (SBV) [9]. One way to limit the impact of RNA viruses and retroviruses is to prevent their replication, and a thorough understanding of the replication and transcription of these pathogens is, therefore, essential.

**Fig. 1:**
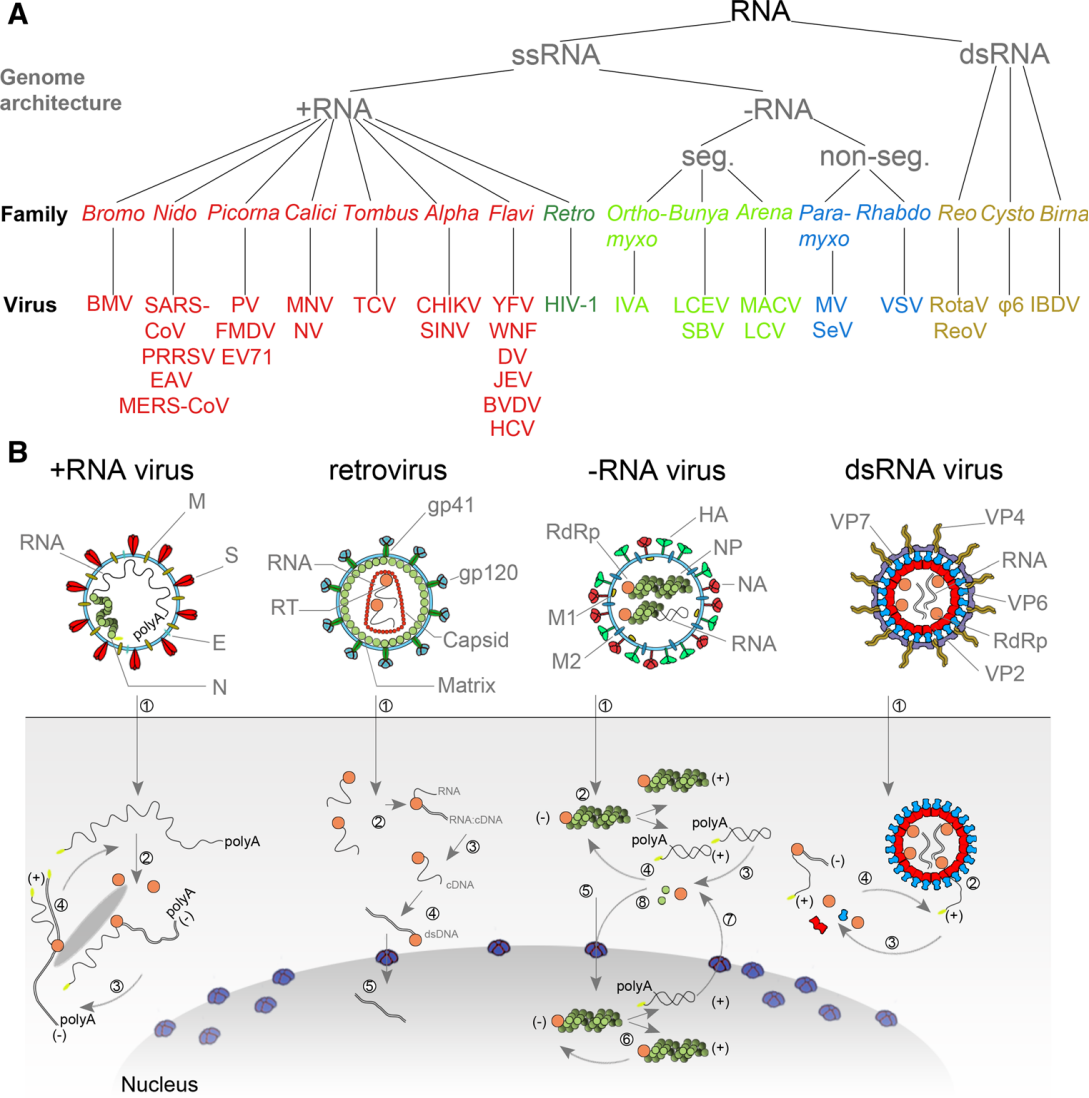
Taxonomy and replication strategies of RNA viruses. **a** Simplified taxonomy of the genome architecture of the RNA viruses described in this review. See main text for used abbreviations. **b** (+*RNA virus*) Infection with a +RNA virus—as exemplified here with a CoV-like virion—releases a single-stranded RNA genome into the cytoplasm (*1*) [81, 173, 174]. (*2*) Translation of the 5′-terminal open-reading frame of the genome produces the viral replicase. (*3*) This multi-enzyme complex includes RdRp activity (*orange*) and associates with intracellular membranes before −RNA synthesis commences. Newly synthesised −RNAs are subsequently used to produce new +RNAs (*4*), which are typically capped (*yellow*) and polyadenylated (polyA). (*Retrovirus*) HIV-1 genomes are packaged as ssRNA in virions. When the ssRNA is released (*1*) a cDNA copy is synthesised by the RT (*2*). The RNA is next degraded by the intrinsic RNase H activity in the RT (*3*) and the single stranded cDNA converted to dsDNA (*4*). The dsDNA is imported in the nucleus (*5*) for integration into the host’s genetic material. (−*RNA virus*) (*1*) As illustrated here with an IAV-like particle, infection with an −RNA virus releases a viral RNA genome that is associated with a viral polymerase (*orange*) and nucleoprotein (*green*). (*2*) In the case of non-segmented −RNA viruses, these complexes support transcription to produce viral mRNAs or cRNAs. (*3*) Viral mRNAs are next translated and new viral proteins complex with cRNAs to synthesise new vRNAs. (*5*) The vRNA-containing complexes of some segmented −RNA viruses are imported into the nucleus of the host cell, where (*6*) the RdRp produces mRNAs or cRNAs. (*7*) mRNAs are transported to the cytoplasm, while cRNAs are bound by new viral proteins to form cRNPs for −RNA synthesis. (*dsRNA virus*) Fully duplexed RNA genomes lack cap and polyA elements. (*1*) The RdRp (*orange*), therefore, transcribes the viral genome inside the capsid of the virion (*blue* and *red*), so viral mRNAs can be (*2*) released into the cytoplasm as illustrated here with a rotavirus-like virion. In the cytoplasm the mRNA is translated (*3*) or replicated by newly synthesised viral RdRps (*4*) [175, 176]

RNA virus genomes can consist of double-stranded RNA (dsRNA) or single-stranded (ssRNA) (Fig. 1a). In turn, the ssRNA viruses can be classified into positive sense (+RNA) and negative sense (−RNA) viruses (Fig. 1a), depending on the translatability of their genetic material. As summarised for four model RNA pathogens in Fig. 1b, all RNA viruses use dedicated replication and transcription strategies to amplify their genetic material. The common denominator of these strategies is a conserved RNA-dependent polymerase domain [10–12].

This review will start with a discussion of the structure of the polymerase domain and its key catalytic residues. Section “Beyond the conserved core” will subsequently focus on the additional domains that are present in polymerase proteins and how they are coordinated. Section “Template recognition, initiation, elongation and regulation” will concentrate on the interactions of the RNA-dependent polymerase with the viral promoter and the initiation of RNA synthesis. Finally, Sect. “Motion and fidelity” will discuss the dynamics and fidelity of RNA-dependent polymerases. A simplified taxonomy of the RNA viruses that will be discussed in these sections is shown in Fig. 1a.

## The RNA-dependent polymerase domain

### Nomenclature and organisation of the conserved elements in the RNA-dependent polymerase domain

**Fig. 2:**
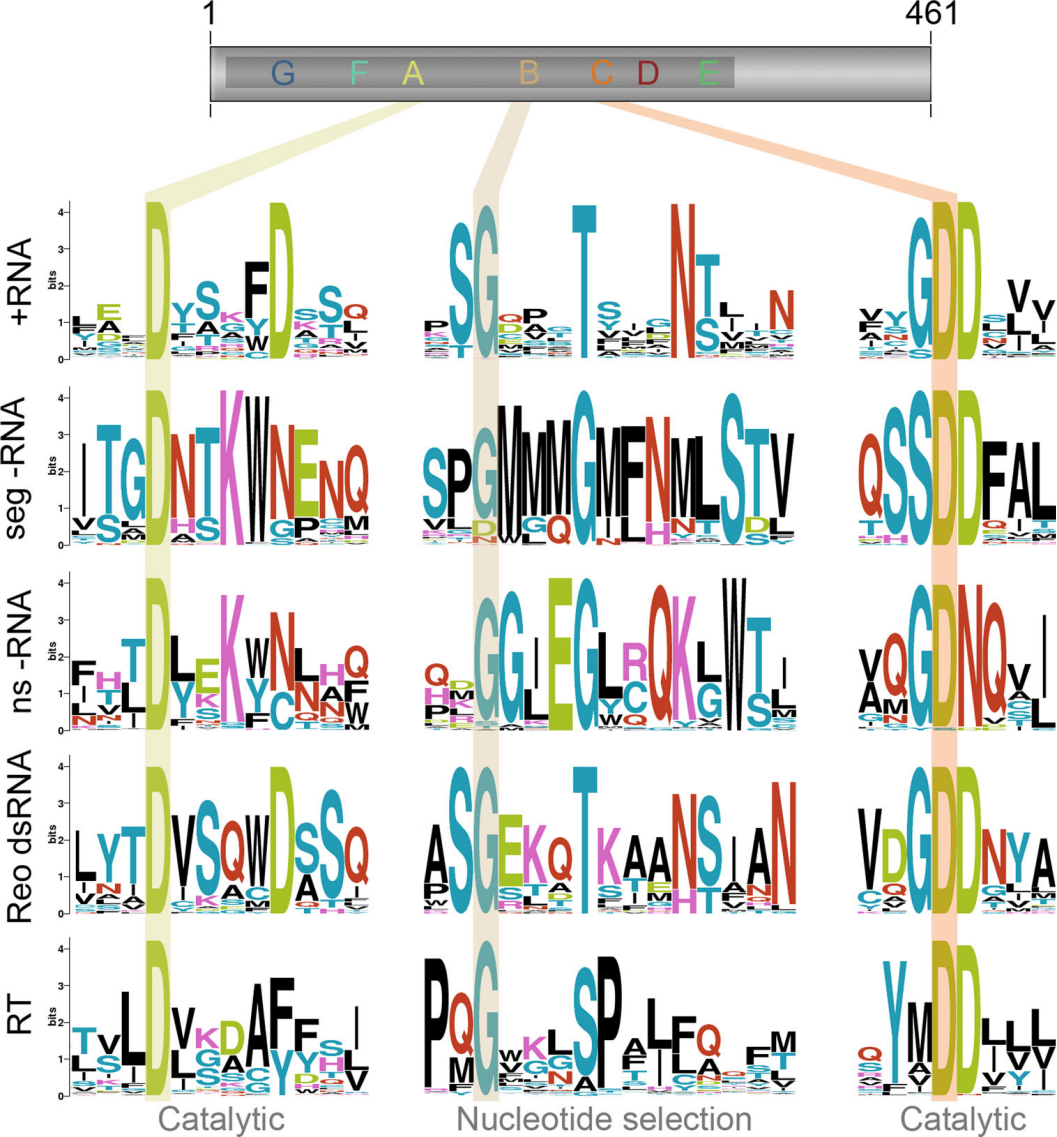
Key conserved residues of the RNA polymerase domain. Motifs A–C reside in the middle of the typical RNA-dependent polymerase domain as shown here in the schematic of the poliovirus 3D^pol^ subunit. They are involved in catalysis and nucleotide selection and the residues involved in these processes are highly conserved. The key residues of these motifs are shaded across the RNA polymerase domains of positive strand RNA viruses (+RNA), segmented negative strand RNA viruses (seg −RNA), non-segmented negative strand RNA viruses (ns −RNA), double strand RNA viruses of the reovirus family (Reo dsRNA), and reverse transcriptases (RT). Sequence logo images were created using prosite accession numbers PDSC50507 and PDOC50878

The RNA-dependent polymerase domain is a member of the superfamily of template-dependent nucleic acid polymerases and typically <400 amino acids in length [10, 11] (Fig. 2). Its sequence is highly variable on average, with some regions showing less than ~10 % conservation [11, 13]. Strong amino acid conservation can be observed, however, in regions that are directly involved in nucleotide selection or catalysis, such as the strictly conserved glycine and aspartates in the centre of the domain [11, 13] (Fig. 2; see Sects. “Function of the conserved aspartates and lysine in the polymerase catalytic site”, “Motifs and structural flexibility are important for nucleotide selection” for details). The prototypic RNA virus RNA polymerase domain harbours seven of such regions or motifs, which are arranged in the order G, F1–3, A, B, C, D and E from amino- to carboxy-terminus [10, 13] (Fig. 2). The only exception to this scheme can be found in the *Infectious bursal disease virus* (IBDV) and related viruses, where motif C is encoded upstream of motif A [14].

**Fig. 3:**
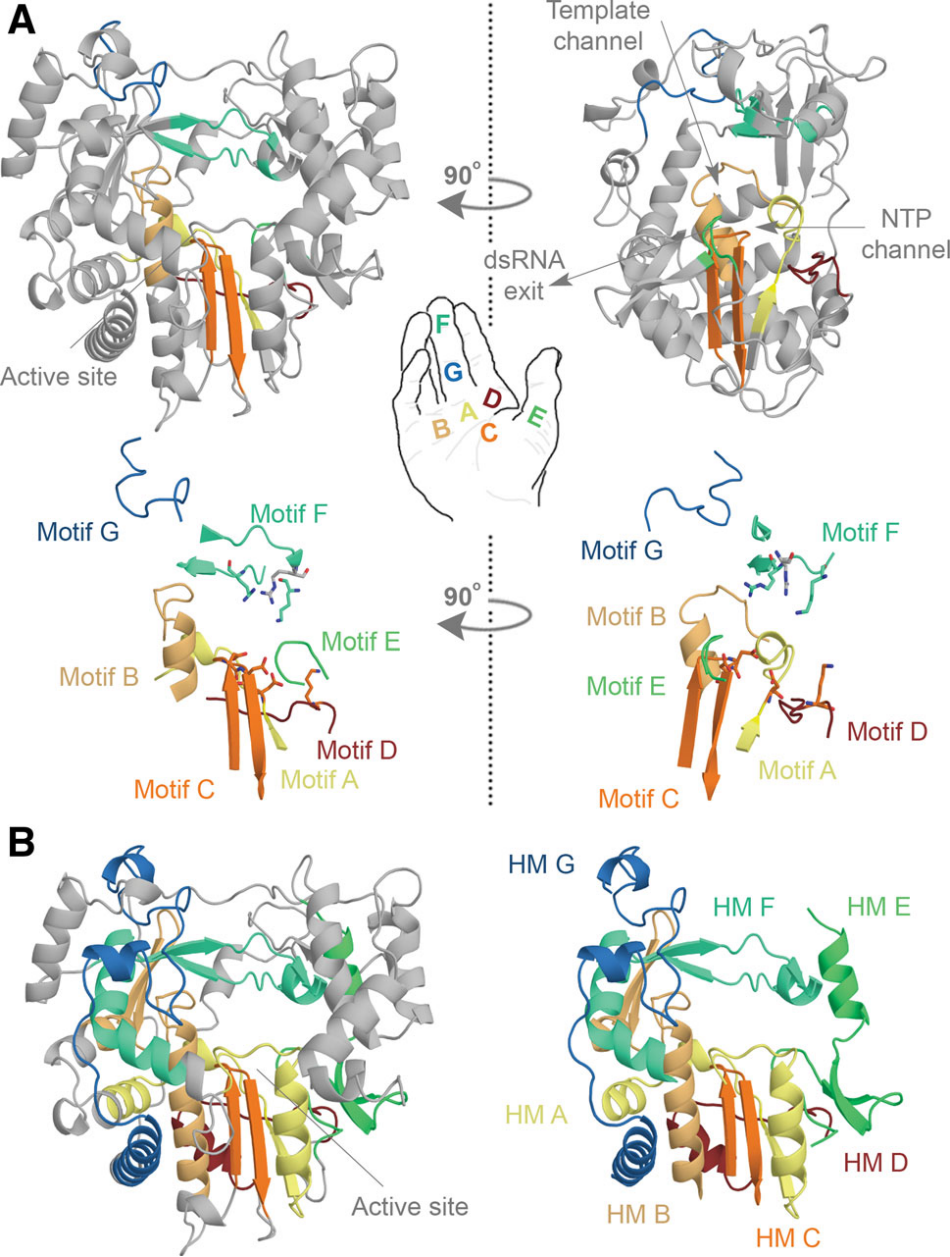
Conserved structural elements in the RNA virus polymerase. **a** Structure of the FMDV RdRp. The motifs A, B, C, D, E, F, and G are colour coded *yellow*, *gold*, *orange*, *red*, *light green*, *aquamarine*, and *blue*. Overall the polymerase structure adopts a shape that resembles a cupped right hand. Herein, motifs A–E lie on the palm, while motif F and G are part of the fingers. In the side-view of the enzyme the location of the template and NTP channels is indicated. **b** Conserved structural elements of the FMDV RdRp. Homomorphs A–G were mapped according to Ref. [15] and colour coded *yellow*, *gold*, *orange*, *red*, *light green*, *aquamarine*, and *blue*, respectively. Images A and B are based on PDB accession 2E9R

**Fig. 4:**
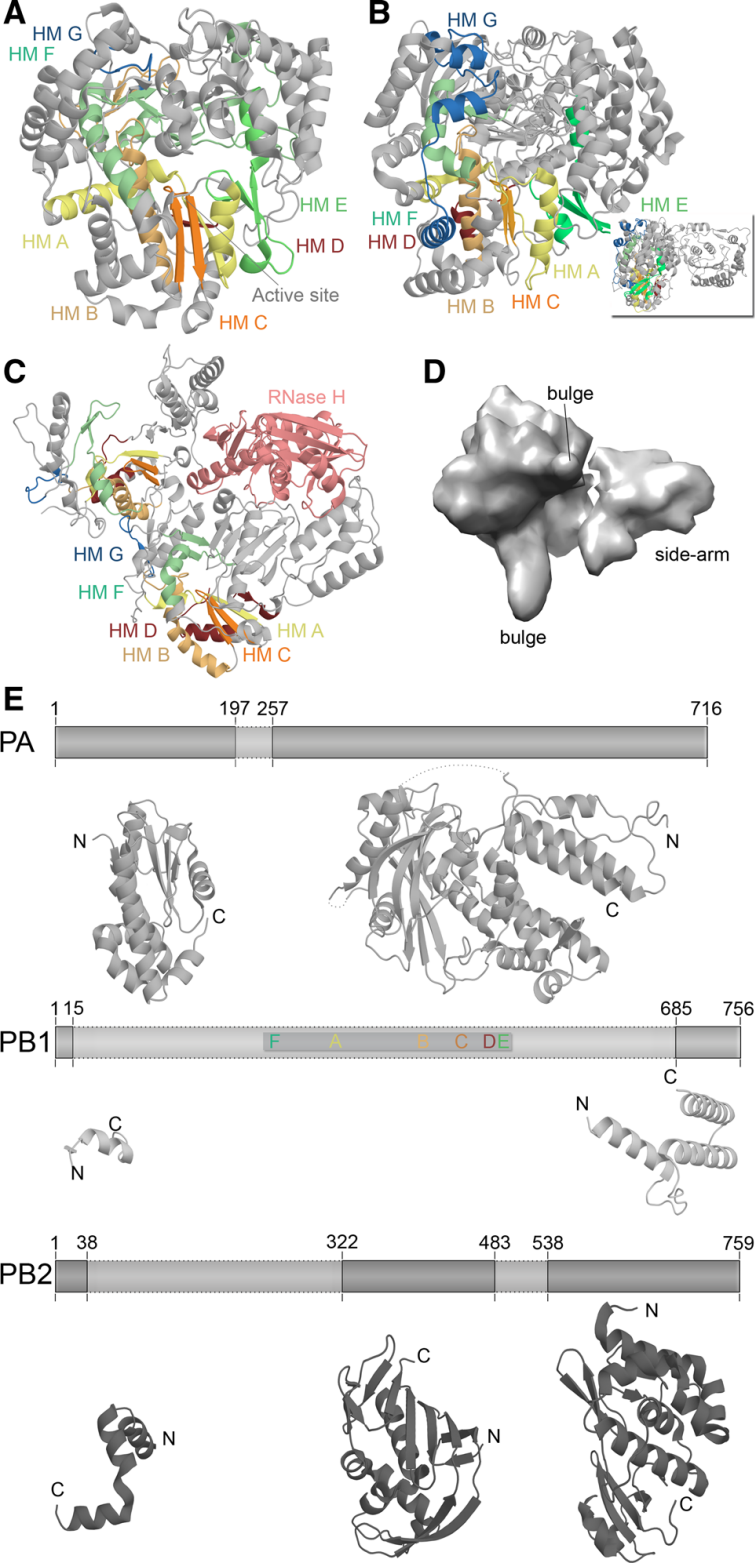
Structural differences among RNA virus polymerases. **a** Structure of the ϕ6 RdRp P2 based on PDB accession 1HI0. **b** Structure of the JEV polymerase based on PBD entry 4K6 M. Inset depicts 90° rotation of polymerase to visualise the N-terminal methyltransferase domain. **c** Structure of the HIV-1 RT based on PDB accession 3V4I. The RT is comprised of the p66 (*left*) and p51 (*right*) protein subunits. Only the p66 subunit has an RNase domain (*pink*). Homomorphs A–G are colour coded *yellow*, *gold*, *orange*, *red*, *light green*, *aquamarine*, and *blue*, respectively in Fig. 4a–c. **d** EM model of the IAV RdRp based on PDBe entry EMD-2213. Structural features were identified by Moeller et al. [79]. **e** The IAV polymerase consists of the subunits PA, PB1, and PB2. Six of the seven canonical RNA-dependent polymerase domains motifs are found in PB1, which are colour coded as in Fig. 3. Presently only significant structural information is available for PA and PB2. Figure based on PBD entries 2VY6, 2W69, 2ZNL, 2ZTT, 3EBJ, and 4CB4

Each of the seven motifs in the RNA polymerase domain adopts a specific and conserved fold [10] (Fig. 3a). However, for most the conservation of the folds extends beyond the regions of sequence similarity into so-called homomorphs [15]. Together, these conserved structural elements make up approximately 75 % of the RNA-dependent polymerase domain [15] (Fig. 3b). In the RdRp structures that are currently available for +RNA, dsRNA, and *Retroviridae* (Fig. 4)—no structures are presently in the PDB for −RNA viruses—these elements define an RNA entry grove at the top of the polymerase, an RNA exit channel at the front, and a channel for the entry of nucleotides at the rear (Fig. 3a) [16–22].

Following the convention for cellular DNA-dependent DNA polymerases (DdDp), the seven motifs and homomorphs are grouped into three subdomains. These subdomains are called fingers, palm and thumb in reference to the polymerase domain’s likeliness to a cupped right hand (Fig. 3a). The finger subdomain loops that interconnect the fingers with the thumb in the RNA-dependent RNA polymerases (RdRps) of +RNA and dsRNA viruses—for instance *Foot and mouth disease virus* (FMDV), *Pseudomonas phage phi6* (ϕ6), and *Japanese encephalitis virus* (JEV) (Fig. 4)—are called the fingertips [23]. This connection creates an overall “closed-hand” conformation that is unique to RdRps and generally not seen in crystal structures of DdDps or reverse transcriptases (RTs) (Fig. 4c).

### Function of the conserved main structural elements

The three subdomains work together to facilitate the binding of RNA and nucleotides (NTPs) [17–20]. The thumb subdomain contains various residues that are involved in RNA binding and these generally pack into the minor groove of the template RNA [20]. In some polymerases, the thumb also contains sequences that protrude into the template channel to help stabilise the initiating NTPs on the ssRNA template (see Sect. “Template recognition, initiation, elongation and regulation” for details on initiation) [17, 18, 24]. Crucially, these protrusions are also able to undergo relatively large conformational rearrangements to facilitate translocation of the template following the first condensation reaction [17, 25, 26]. The other residues of the thumb subdomain contribute to the formation of the NTP tunnel, which sits at an ~110° angle with the template channel (Fig. 3a). The cavity is lined with positively charged amino acids [17–20], though charge interactions are likely not sufficient to guide NTPs into the interior. Indeed, molecular dynamics (MD) simulations have shown that the residues of the NTP channel can also explore a relatively large amount of space [27], which may allow the RdRp to actively “pump” NTPs down the channel [27].

**Fig. 5:**
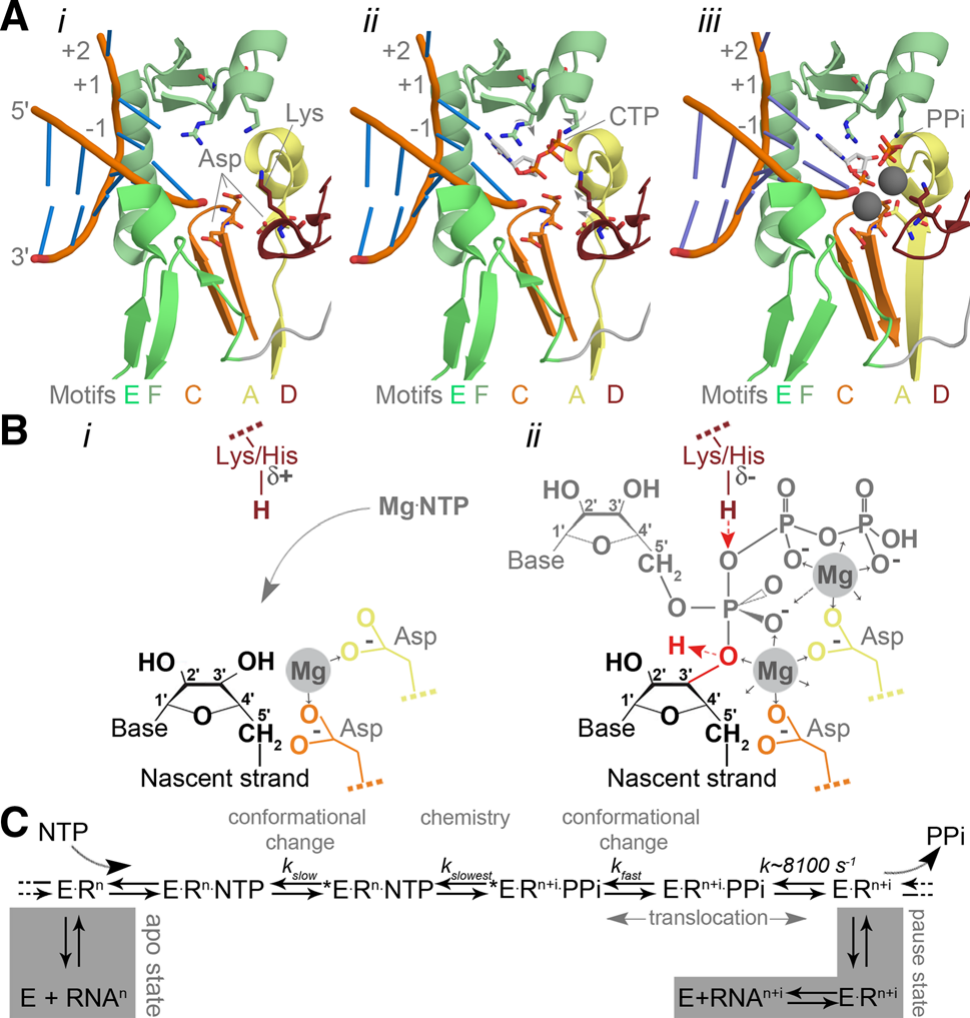
Catalysis in the RNA virus polymerase active site. **a** Structure of the PV active site as it moves from a native state or elongation complex (*i*) to an open complex (*ii*), and a closed complex (*iii*). The closed complex depicted here shows the active site after catalysis. Highlighted are the metal-binding aspartates of motifs A and C, and the lysine of motif D that acts as general acid. Colour coding by motif as in Fig. 3. Image based on PDB accessions 3Ol6, 3OLB, and 3OL7. **b** Schematic presentation of the RdRp active site. The aspartates (Asp) of motif A (*yellow*) and C (*orange*) bind divalent metal ions (marked Mg and *shaded grey*), which are used to coordinate the formation of a new phosphodiester bond at the 3′-OH (*red* in panel ii) of the nascent strand (*yellow*). The general acid (*red* Lys/His in panel ii) is positioned near the β-phosphate of the incoming NTP to protonate the PP_i_ leaving group. **c** Simplified schematic of the kinetic steps of RNA polymerases. *Asterisk* indicates closed complex

The fingers subdomain residues mainly pack into the major groove of the RNA template. Furthermore, upon entry of the template they are able to unstack the single strand at position +3 [20] (Fig. 5a). The non-conserved structural elements of the fingers subdomain play a role in RNA binding as well [17, 18, 20]. In particular, the fingertips of dsRNA and some +RNA virus RdRps allow the polymerase to “hold” the template without extensive conformational changes [18, 28]. The variations and extensions in the fingers subdomain have also been shown to play roles in protein–protein interactions, phosphorylation, oligomerisation, and nuclear import [24, 29–31]. In contrast, the HIV-1 RT lacks such extensions and adopts a more “open-hand” or U-shaped binding cleft [21] (Fig. 4c). As a consequence, the RT structure must rearrange its subdomains to coordinate the binding of the template, nascent strand, and incoming dNTPs.

The NTP and template entry channels meet at the palm subdomain (Fig. 3a). This is a structure that is comprised of a central, partially formed three-stranded β-sheet, which is also present in RNA-recognition motifs (RRMs). The relative movement of these strands within the RRM is vital to catalysis and dependent on NTP binding. Only when a correct NTP binds can motif A and motif C align and the RRM fold be completed [20] (Fig. 5a). This catalytically competent conformation of the active site is often referred to as the closed complex (not to be confused with the “closed-hand”, which refers to the overall structure of the RdRp) [20].

### Function of the conserved aspartates and lysine in the polymerase catalytic site

The palm subdomain motifs A, C and D are the most essential elements in the RNA polymerase domain. This is particularly evident from the strict conservation of the N-terminal aspartates in the Dx_4–5_D and xDD consensus sequences (here ‘*x*’ represents any residue) of motifs A and C (Fig. 2). Mutational analysis of these residues has shown that their absence abolishes or greatly alters RNA-dependent polymerase activity in vitro and in vivo [32–36]. This is in line with structural studies showing that the N-terminal aspartates coordinate two divalent metal ions that are crucial for polymerase function [17–20] (Fig. 5a, B). In contrast, motif D typically contains a sole lysine or histidine as signature amino acid [37, 38]. This residue is likely protonated in the environment of the active site and its mutation strongly reduces the activity of the RNA polymerase [37, 39–41]. Moreover, an arginine to lysine mutation at motif D’s position in the polymerase of a *Hepatitis C virus* (HCV) strain increases the activity of this RdRp [42].

The polymerase reaction creates new phosphodiester bonds between NTPs using RNA as template. The NTP substrates involved in this reaction are coordinated by two metal ions, which are bound by the conserved aspartates of motifs A and C (Fig. 5a, panel i). They also position the NTP’s triphosphate optimally for attack by the sugar moiety of the nascent strand once its 3′ carbon has lost a proton [17–20] (Fig. 5b). The N-terminal aspartate of motif C thus uses a metal ion to fix the α-phosphate of the incoming NTP and reduce the pK_a_ of the nascent RNA’s 3′-OH to facilitate the attack (Fig. 5b, panel ii) [43]. The amino-terminal carboxylate of motif A, on the other hand, stabilises the β- and γ-phosphates with its divalent metal as well as the pentacovalent (phosphorane) intermediate that forms during catalysis (Fig. 5b, panel ii). Structural and biochemical analyses have shown that the formation of this transient structure is dependent on the attack of the NTP’s α-phosphate by the 3′-OH, which is often the rate-limiting catalytic step in NTP condensation [37, 43] (Fig. 5b).

Motif D’s lysine or histidine assists the N-terminal aspartate of motif A in coordinating the β-phosphate of the incoming NTP, analogous to the trigger-loop in DdDps [38, 44]. However, when the positively charged side chain of motif D approaches the β-phosphate, it can protonate the PP_i_ leaving group as well (Fig. 5b, panel ii). This second protonation step in the active site is not essential for the polymerase reaction, since catalysis can still take place when motif D’s lysine has been mutated to a residue with a different pK_a_. The polymerase reaction rate will nevertheless be 50- to 1,000-fold higher when a lysine or histidine is present [38, 45]. Recent data even suggests that motif D can coordinate the export of the PP_i_ group from the active site once catalysis has taken place [45], thereby triggering the translocation of the RNA.

### Motifs and structural flexibility are important for nucleotide selection

Biochemical and structural analyses have shown that polymerase catalysis is a multi-step process that includes at least one NTP recognition event (Fig. 5C). This step must ensure that the catalytic residues and the substrate are not placed in a catalytically competent position before a correct NTP is bound. In part, this is achieved by motif D, which needs to bind the β-phosphate of the incoming NTP to trigger active site closure (Fig. 5a, b). Crucially, due to its highly flexible nature it can only adopt this position when a correct NTP is bound [44–46] and it is now believed that getting motif D near the β-phosphate is the second most rate-limiting step (*k*_slow_) in the RNA polymerase catalytic cycle after chemistry (Fig. 5c).

Motif D’s conformational change is thus the final checkpoint in the NTP selection process and other motifs facilitate the steps that lead up to this event. Subtle movements in motif B, for instance, help position the template and the incoming NTP via direct interactions between the base and conserved residues in the N-terminus of motif B [20, 47]. Motif F, on the other hand, contributes several evenly spaced, positively charged amino acids that coordinate the negatively charged triphosphate of the NTP (Fig. 5a) [17–20]. In the case of HIV-1 RT, the binding of a dNTP also triggers a rotation of the motif F-containing homomorph towards the polymerase active site [21]. As this motion creates a relatively tight fit between active site, dNTP, and the basic residues of motif F, only the binding of the correct dNTP can trigger it [21].

In +RNA RdRps, selection between NTPs and dNTPs is supported by motif B’s conserved asparagine and the C-terminal aspartate of motif A (Fig. 2). Both form a crucial hydrogen bond with the 2′-OH of the incoming NTP [48] and mutation of conserved motif B residues cripples polymerase activity [42, 49]. In dsRNA RdRps, residues with equivalent functions are an aspartate that is positioned just N-terminal of motif A and a conserved serine in motif B [17]. In the HIV-1 RT, on the other hand, these residues are a tyrosine just N-terminal of motif A and, most frequently, a phenylalanine at the same position as the +RNA virus asparagine, since their side chains are better suited to facilitate a hydrophobic interaction with the 2′-H of the incoming dNTP [21].

## Beyond the conserved core

### Additional enzymatic domains

**Fig. 6:**
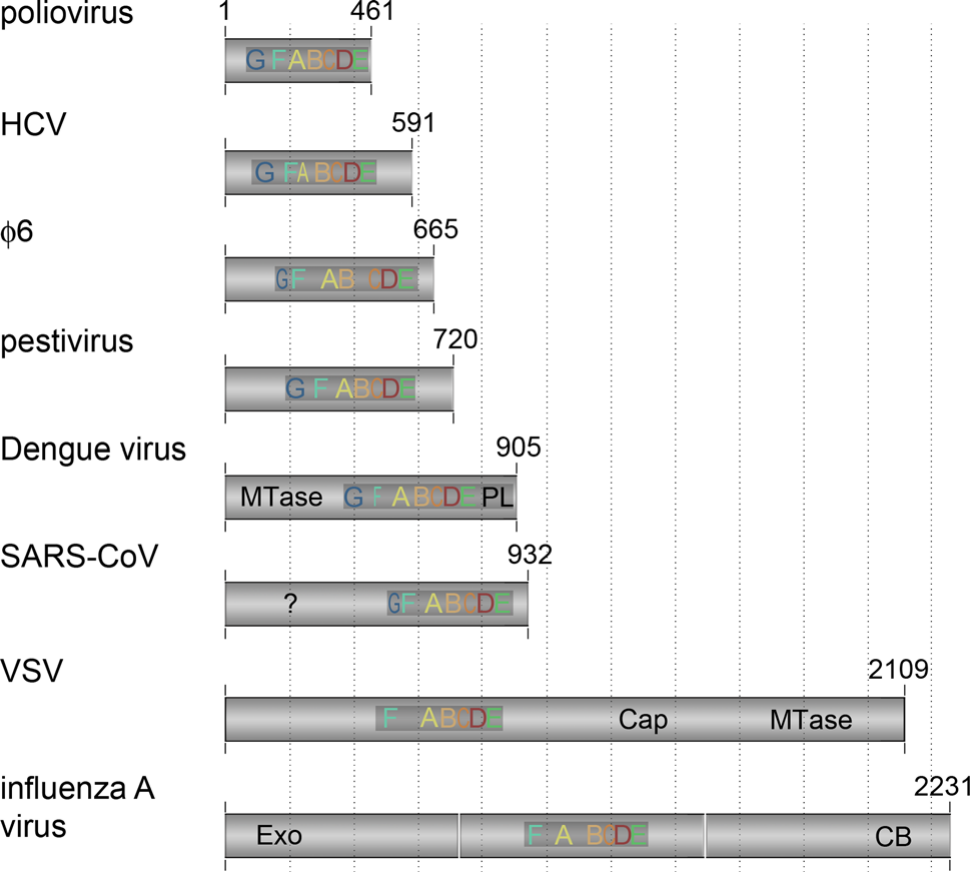
Sizes and additional domains of RNA virus polymerases. The RNA-dependent polymerase domain consists of approximately 400 amino acids. The size of the rest of the subunit varies significantly among RNA-dependent polymerases, as does the conservation of motifs F and G. The additional sequences of the polymerase subunit often contain functional domains, such as a methyltransferase (MTase), exonuclease (Exo), capping domain (Cap), or cap-binding domain (CB). The SAR-CoV RdRp has an additional N-terminal domain that is conserved, but to which no function has been ascribed yet (?). Figure based on alignments from references [15, 39, 83, 177, 178]

Additional domains frequently flank the polymerase domain (Fig. 6). Good examples are the polymerase subunits of the *Bunya*- and *Arenaviridae*, which contain both an N-terminal endonuclease domain as well as a C-terminal polymerase domain [50, 51]. An even more striking example is the *Vesicular stomatitis virus* (VSV) RdRp (Fig. 6). In electron microscopy (EM) images, this enzyme appears as a central density that is surrounded by globular appendages [52], which harbour enzymatic activities that are required for viral mRNA5’ cap synthesis: a 2′-O-methyltransferase (MTase), a guanine-N7-MTase, and a polyribonucleotidyl-transferase (PRNTase) [53, 54]. Such activities can also be found in the RdRps of *Flavivirus* genus members, albeit with a guanylyltransferase (GTase) instead of a PRNTase [55, 56]. As is shown in the inset of Fig. 4b, the MTase of NS5 is connected to the polymerase domain via a flexible linker. It is likely that the discrete domains of other RNA virus polymerases are connected via such linker sequences as well [57]. The benefit of such flexible attachments is that it allows domains to work *in trans* or even separately from the polymerase domain, as was demonstrated for the MTase and endonuclease domains of the *Sendai virus* (SeV) and *Lymphocytic choriomeningitis virus* (LCV) RdRps [50, 58]. Moreover, accessory domains can sometimes also be deleted from the polymerase subunit without disrupting the activity of the polymerase domain [24, 41].

**Fig. 7:**
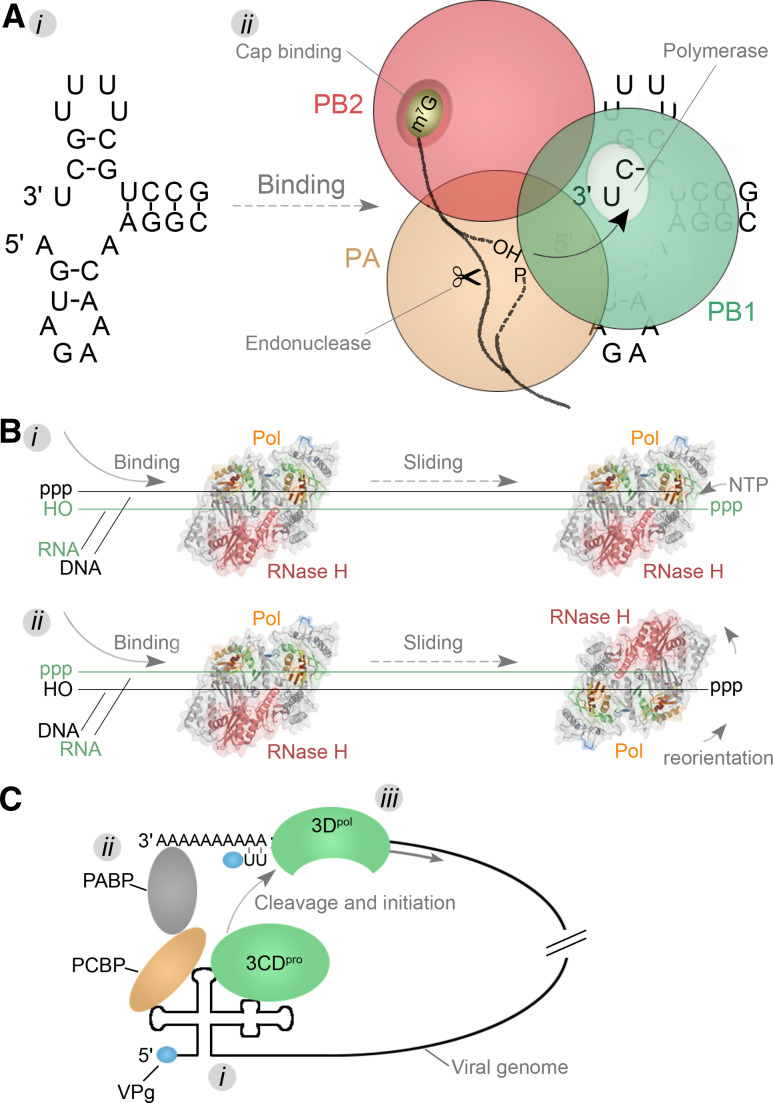
Template recognition mechanisms. **a** *i* The IAV RNA promoter forms through hybridization of the terminal ends of a viral genome segment. *ii* The structure is next bound by the heterotrimeric RdRp complex that consists of subunits PB1 (*green*), PB2 (*red*), and PA (*orange*). During transcription, the PB2 subunit binds the cap of pre-mRNAs. These pre-mRNAs are next cleaved by the PA subunit and transferred to the PB1 subunit. Here nucleotide condensation takes place on the 3′-OH of the capped primers. **b** The HIV-1 RT is comprised of the subunits p66 and p51, of which only the p66 subunit has an active polymerase (colour coded as Fig. 4) and RNase domain (*pink*). The enzyme can bind to the template in a random orientation and switch between the activities by tumbling around its axis once a polymerase (*i*) or RNase (*ii*) substrate has been encountered. **c** A model for the circularisation of the PV genome. First viral proteins and the 3CD^pro^ cleavage intermediate bind to conserved 5′ structures on the genome (*i*). Next a protein bridge is formed that brings together the 5′ and 3′-ends of the genome (*ii*). This step subsequently ensures that the viral protease is activated and the viral polymerase 3D^pol^ is released in close proximity to the 3′-end of the viral genome. Here it can initiate -RNA synthesis using VPg as protein primer

The IAV genome, in contrast, encodes an RdRp in which its multiple activities are separated out into three individual polypeptides, called PA, PB1, and PB2 (Figs. 4, 6). The three subunits are encoded by three negatively stranded genome segments (vRNAs), which are replicated and transcribed in the nucleus of the host in complexes (vRNPs) of heterotrimeric polymerase and a helical assembly of nucleoprotein (NP) monomers (Fig. 1). The RdRp motifs F–E are housed in PB1 [59, 60], while a metal-dependent endonuclease site is present in PA and a cap-binding domain resides in PB2 (Fig. 6). It is now widely accepted that IAV RNA synthesis initiates replication de novo, whereas transcription initiates in a primer-dependent manner (see Sect. “Template recognition, initiation, elongation and regulation” for details). In the latter scenario, a 5′ capped pre-mRNA molecule is bound by the PB2 subunit (Fig. 7a). The pre-mRNA is then cleaved by the PA subunit 9–15 nt downstream of the cap and moved into the PB1 active site for template-dependent extension [59, 60]. How the molecular hand-off is performed and the 3′-OH of the capped primer transferred to PB1 is presently unknown.

Such a hand-off between enzymatic domains has been studied for the HIV-1 RT. This enzyme is effectively a dual-polymerase complex that consists of the viral p66 subunit, which contains an RNase H domain and a polymerase domain [61], and its cleavage product p51, which lacks the former domain [62] (Fig. 4c). During retrotranscription, the two active sites in the enzyme must catalyse a multi-step process, including (1) RNA-templated cDNA synthesis using a lys3 tRNA as primer, (2) dsDNA synthesis using the cDNA strand as template, and (3) hydrolysis of the incorporated lys3 RNA primer sequences [63] (Fig. 1b). Although the two enzymatic activities of RT can work *in trans* [64], fluorescence resonance energy transfer (FRET) assays demonstrated that the enzyme can flip along its axis and switch between the activities (Fig. 7b). This principle allows RNA hydrolysis and NTP condensation to effectively work *in cis*, irrespective of how RT initially bound [65, 66]. Interestingly, such an apparent dissociation-rebinding event is in contrast with the intramolecular translocation of the nascent strand between the polymerase and exonuclease sites in DdDps [67, 68]. It will, therefore, be interesting to see how other viral RNA-dependent polymerases orchestrate a hand-off between active sites.

### Multimerisation

Oligomerisation of RdRps has been reported for *Poliovirus* (PV), FMDV, HCV, *Norovirus* (NV), and IBDV enzymes. It can result in cooperative template binding, lattice formation, and a stimulation of viral RNA polymerase activity in vitro [69–72], which in HCV appears to be specific for initiation [73]. Also inactive RdRps can stimulate activity and participate in array formation, which led to the hypothesis that multimerisation evolved to stabilise polymerases [69]. Mutagenesis studies suggest that the dimerisation of the HCV RdRp is mediated by the thumb domain [74], whereas in PV 3D^pol^ residues in N-terminal part of the polymerase domain and two aspartates of the so-called interface I—an interaction interface between the thumb subdomain of one RdRp and the palm of another—play a role [46, 69]. Mutation of the interacting residues also results in viral growth defects [75].

Polymerase multimerisation may also play a role in IAV infections [76]. In the replication cycle of this −RNA virus, the viral RdRp synthesises new vRNAs through a complementary +RNA (cRNA) intermediate, which is bound by newly produced viral RdRp and NP to form cRNPs (Fig. 1). Crucially, during the synthesis of vRNAs, the cRNP can be stimulated by both active as well as inactive secondary RdRps [77], which suggests that cooperation takes place. In contrast, experiments with vRNPs suggest that secondary −RNA virus polymerases are *trans*-acting and only able to complement the activity of the resident RdRp during the synthesis of cRNA copies [78, 79]. Curiously, no evidence has been found for either cooperation or *trans*-acting activity during transcription, which suggests that cap-snatching, mRNA synthesis, and polyadenylation all occur *in cis* [78] and putatively without multimerisation. It is presently not known how any of the two replication-specific interactions between the IAV polymerases are mediated.

In contrast to the above examples of multimerisation, which all involve multiple copies of essentially the same polypeptide, the positive-stranded CoVs have been hypothesised to use two distinct polymerase activities for their RNA synthesis [80]. The CoVs are well known for their uniquely large ~30-kb +RNA genomes, of which approximately two-thirds code for numerous viral replicase functions that associate with cellular membranes upon translation [81] (Fig. 1). Nearly three decades ago, bioinformatics analyses predicted that a canonical RNA polymerase domain resided among these replicase functions in a 932 amino acid-long mature protein [82] (Fig. 6). Since then, biochemical experiments of the CoV replicase protein have not only confirmed this prediction [33, 83], but also revealed a non-canonical, multimeric RdRp that is able to recognise the 3′ terminus of the viral genome [84, 85]. It has been proposed that these two enzymes may cooperate to improve or to achieve better control over initiation and elongation, putatively functioning as primase and primer-dependent polymerase [80].

## Template recognition, initiation, elongation and regulation

### Template recognition

The association between the heterotrimeric IAV polymerase and viral RNA can be solely achieved through interactions with a partially complementary RNA promoter, which consists of the conserved 5′ and 3′ terminal sequences that are present at each genome segment or complementary genome segment [86]. A similar sequence element and one that is also based on complementarity can be found in the genomic ends of the genomes of the *Arena*- and *Bunyaviridae* [87]. It is currently understood that the IAV polymerase first associates with the 5′ end of the promoter, before the 3′ terminus is bound [88–90]. Mutagenesis data suggest that the binding process creates structural rearrangements in the promoter, changing its conformation to a cork-screw-like structure (Fig. 7a). It is assumed that conformation directs the viral transcription and replication activity of the polymerase [91–93]. Other experiments have shown that the promoter is also vital for the packaging of viral genome segments into virions [94] and potentially in overcoming host restrictions [95].

In *Flaviviridae*, RdRp recruitment and binding are partially guided by circularisation of the genome. RNA structures, such as the stem-loop/promoter structure in the 5′ UTR of the genome, are strongly involved in the initiation of replication as well [96, 97]. The involvement of the 5′ UTR can be explained by the interaction it establishes with the conserved elements of the 3′ UTR, thereby circularising the genome akin to the segmented −RNA viruses. Such a long-range interaction can also effectively position the conserved 3′-terminal sequence 5′-CU^OH^ opposite the 5′-terminal end [96, 98–100], which would facilitate recognition of the 3′-terminal end and minimise initiation on non-viral templates [101].

In contrast, the plant virus *Brome mosaic virus* (BMV) requires an additional viral protein to bring the viral 2a polymerase and genome together. The interaction is mediated by protein 1a, which can bind both RdRp and conserved so-called box B motifs in the viral RNA [102]. The helicase domain of 1a may also stimulate RdRp initiation [103], which is believed to take place on a conserved 3′-terminal cytosine during replication and an internal cytosine during subgenomic transcription [104]. The involvement of conserved (3′-terminal) cytosine residues appears to be a recurring theme in RNA virus replication or transcription, as they are also used for initiation by the *Reovirus* (ReoV), *Machupo virus* (MACV), and the *Equine arteritis virus* (EAV) [17, 105, 106].

In infections with members of the *Picornaviridae,* both circularisation and the use of viral protein factors can be observed (Fig. 7c). Here the 5′ structures in the genome present a binding platform for viral and cellular proteins. The most downstream of the these 5′ elements is currently believed to serve as promoter for both +RNA and −RNA synthesis [107] (Fig. 7c, marked i), while the upstream cloverleaf structure functions as binding site for the cellular poly(rC)-binding protein (PCBP) and the viral 3CD^pro^ polyprotein cleavage intermediate [108–110]. To setup PV 3D^pol^ for RNA synthesis, the various proteins already associated with the viral genome must first bind the C-terminal part of the cellular poly(A) binding protein (PABP) [111], which effectively circularises the viral genome (Fig. 7c, marked ii). In turn, the circularisation stimulates the self-cleavage of the 3CD^pro^ precursor and thereby the activation of the RdRp 3D^pol^ (Fig. 7c, marked iii), whose activity is inhibited as long as its N-terminus has not been processed [112, 113]. Typically the RdRp next initiates on the *cis*-replication element (CRE) in the viral genome [114].

### De novo initiation

**Fig. 8:**
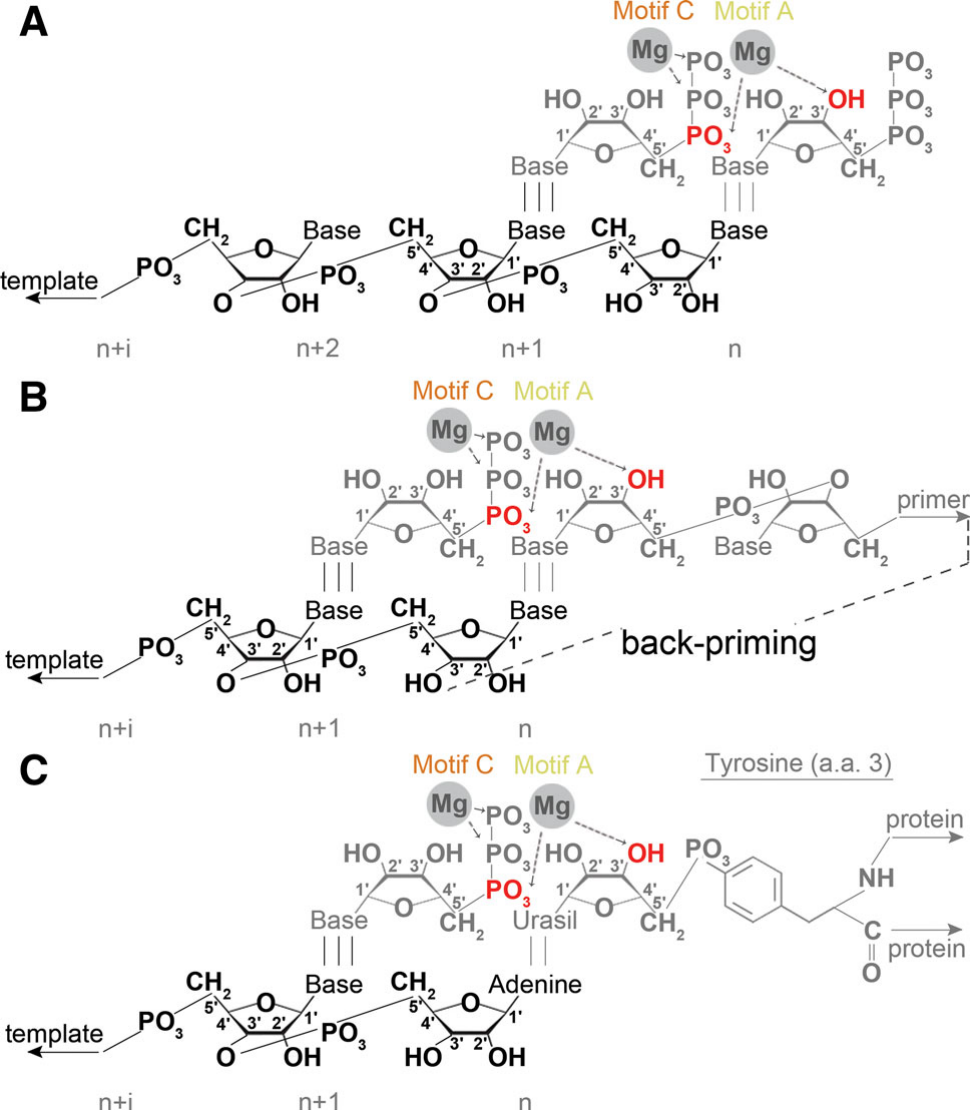
Modes of initiation. schematic presentation of **a** de novo initiation, **b** primer-dependent initiation, and **c** protein-dependent initiation. In the latter scenario, the tyrosine residue that is present at position 3 of VPg is used as primer

In spite of the wide range of replication strategies used by RNA viruses, their polymerases can use only two mechanisms to initiate the synthesis of RNA: *de novo* initiation or primer-dependent initiation (Fig. 8). The *de novo* initiation mechanism can be subdivided in 3′ terminal initiation or internal initiation. Some polymerases can also use a combination of both which is called prime-realign initiation [91, 115]. The advantage of the latter strategy is that no genetic information is lost, since internal residues are better protected from cellular exonucleases.

During *de novo* initiation, an initiating nucleotide serves as primer for a second nucleotide. These two NTPs are base paired with positions +1 and +2 of the template, respectively (Fig. 8a). Since base pairing with the template is not sufficient to hold both NTPs correctly positioned for catalysis, polymerases can also employ a number of techniques to stabilise the interaction. The ReoV RdRp λ3 uses a loop that protrudes from the palm domain to bind the negative triphosphate backbone of the NTP at position +1, which is typically GTP [17]. Other residues in the active site, most notably an arginine (R518) in motif F and a serine (S682) in motif B, form hydrogen bonds with the carbonyl and NH group of this GTP [17], while a conserved glutamine in motif C binds the 2′-OH of the GTP’s ribose [17]. Together these interactions not only stabilise the initiating GTP, but also provide nucleoside selection to keep the 3′-terminal cytosine present in both the plus and minus strand of ReoV RNAs preserved.

**Fig. 9:**
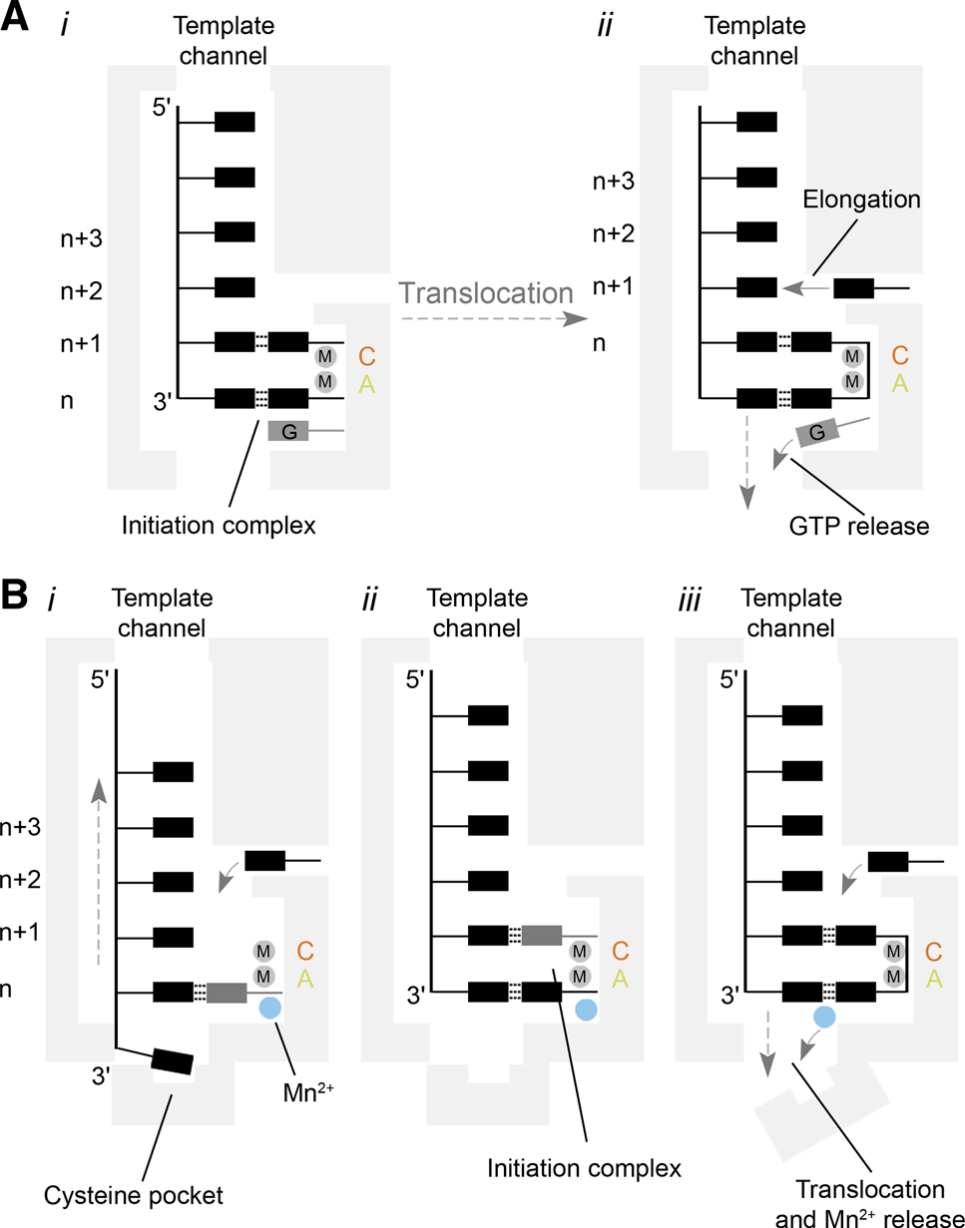
Models for template recognition and de novo initiation by the BVDV and ϕ6 polymerases. **a** Model of de novo RNA synthesis by a BVDV RdRp. The RdRp binds to the 3′-end of the viral genome (*i*). Then the template, initiating NTPs, and an additional GTP form an initiation complex that is stabilised by the closed C-terminal loop. After the first polymerase reaction, the GTP is released by opening of the C-terminal loop and translocation of the template–nascent strand duplex (*ii*). A new NTP can enter the active site via the NTP channel to start elongation. **b** Simplified model of de novo RNA synthesis by the ϕ6 RdRp. Template recognition occurs through the binding of the 3′-cytosine of the genome to a pyrimidine specificity pocket (*i*). At the same time, a nucleotide base-pairs with the second base of the template. Its triphosphate group is stabilised by a manganese ion that is coordinated by the palm domain. The 3′-cytosine next translocates to the n-position to base-pair with a second NTP (*ii*). After nucleotide condensation, the manganese ion triggers a rearrangement of the C-terminal and is subsequently released (*iii*). In contrast to the GTP in the NS5 RdRp, the manganese ion must be rebound for subsequent elongation

Analogously, in some +RNA virus RdRps a short polypeptide loop can protrude from the thumb domain into the active site to perform these functions [24, 25, 116]. In the *Bovine viral diarrhoea virus* (BVDV) RdRp, this so-called primer-loop is assisted with a non-templated GTP molecule (Fig. 9A). Positioned in a −1 position ~6 Å away from the catalytic site, the additional GTP creates a stacking platform for NTPs that bind at positions +1 and +2 [24] (Fig. 9a). In BVDV, the N1 and N2 of the guanine are bound by the carbonyl of a motif A threonine, while the hydrophobic proline and leucine residues in motif A and the thumb subdomain stabilise the guanine base. In addition, positively charged residues in motif E stabilise the triphosphate backbone of the GTP [24]. GTP also plays a role in *de novo* initiation of the JEV polymerase, as demonstrated by Surana et al. [117]. However, in contrast to its role in BVDV, the GTP here binds in an orientation that can prevent template binding. Addition of manganese can resolve this blocked pre-initiation state and it is possible that this mechanism evolved to limit non-templated RNA synthesis [117].

The RdRp of ϕ6 stabilises the initiating nucleotides with an additional, small C-terminal domain in at least three ways. First, it blocks the RNA exit channel, which fixes the template strand, not unlike the priming loop in other RdRps (Fig. 9b) [18]. A Mn^2+^ that is bound ~6 Å away from the catalytic site by residues in the palm subdomain also plays a role in template binding [18]. Second, it provides a small, pyrimidine-sized specificity pocket in which a glutamate hydrogen bonds with the NH_2_ of the 3′-terminal cytosine that is present in all ϕ6 genome segments (Fig. 9b, panel i). This also means that an incoming NTP first base pairs with position +2 of the template and that position +1 can be occupied when the template ratchets back from the cytosine pocket (Fig. 9b, panel ii) [18]. Third and last, the C-terminal domain contains a tyrosine (or tryptophan) on which the initiating GTP can stack and stably base pair with the conserved 3′-terminal C at the +1 site [18]. Crucially, after the formation of the dinucleotide, the C-terminal domain has to be displaced to allow RNA translocation and current evidence suggests that the non-catalytic Mn^2+^ is also involved in this step [26]. A non-catalytic Mn^2+^ has also been observed in other RdRps [118].

The RdRps encoded by some -RNA viruses, including the heterotrimeric RdRp of IAV, can perform both *de novo* initiation as well as primer-dependent initiation. The de novo mechanism is used to start the synthesis of cRNAs from vRNAs (Fig. 1). In turn, these cRNAs can be used as template for the production of new vRNAs. This latter process utilises a prime-realign mechanism that depends on high concentrations of initiating NTPs [91]. These initiating NTPs base pair with positions +4 and +5 of the cRNA 3′ terminus [91] and then translocate to bases +1 and +2 after the first nucleotide condensation as dinucleotide pppApG. Although it has been shown that the structure of the viral promoter and its sequence play an important role in this mechanism [91], it is presently unknown which residues of the −RNA virus RdRp are involved in stabilising the first nucleotide bond. It is also unknown how the pppApG is transferred to the 3′ terminus of the cRNA template.

### Primer-dependent initiation

Primer-dependent initiation can be categorised in oligonucleotide-primed, protein-primed, and back-primed initiation, in which the 3′ terminus of the template forms a small hairpin (Fig. 8b, c). Since the use of a primed template requires a template channel that can accommodate several base pairs of A-form dsRNA, the *Corona*- and *Picornaviridae* polymerases such as PV 3D^pol^ and SARS-CoV nsp12 lack the palm or thumb subdomain protrusions found in most *Flaviviridae* and dsRNA viruses [33, 83, 119, 120]. Consistent with this observation, it was found that the HCV and ϕ6 polymerases can be changed into primer-dependent polymerases by mutating or deleting their *de novo* initiation platforms [121–123].

Although the PV polymerase 3D^pol^ can initiate on partial dsRNA, it uses a protein primer in vivo. Following its activation through the self-cleavage of 3CD (Fig. 7c), 3D^pol^ uridylates the PV protein VPg on the tyrosine 3 (Fig. 8c) using a back-slide mechanism on adenosine residues [124]. This creates a VPg(pUpU^OH^) molecule that can hybridise to the viral 3′ polyA tail and prime the synthesis of a complementary strand (Fig. 7c). A study of the related FMDV RdRp has shown that the terminal uridylate of this product can enter the active site and prime a duplication of the genomic 3′ end [125]. Furthermore, VPg(pUpU^OH^) can anneal to the 3′-terminal adenosine residues of the negative strand and thereby prime +RNA synthesis as well [107, 126, 127].

The *Orthomyxo*-, *Arena*-, and *Bunyaviridae* use a primer-dependent initiation mechanism for transcription. In IAV, this process begins with the binding of a 5′ capped pre-mRNA molecule by the PB2 subunit (Fig. 7a, panel ii) [128, 129]. A crystal structure of the IAV PB2 subunit with cap analogue m^7^GTP demonstrated that its structure is similar though different from other known cap-binding proteins [128]. It coordinates binding via histidine and lysine residues that hydrogen bond with the 2′ OH of the ribose, a histidine and asparagine that coordinate the phosphates, and histidine, phenylalanine and lysine residues that sandwich the m7-methylated guanine base [128, 130]. After cap binding, the PA subunit cleaves the pre-mRNA 9–15 nt downstream of the cap with a strong preference for cleaving 3′ of guanine bases [59, 131] (Fig. 7a, panel ii). The endonuclease domain of the L-protein of *Bunyaviridae* shows a strong similarity to the IAV PA subunit [50, 51]. How the 3′-OH of the capped primer is transferred to the polymerase domain in the L-proteins of the *Arena*- and *Bunyaviridae* and the PB1 active site in IAV is presently unknown.

## Motion and fidelity

### Rates of motion within the polymerase structure

Several steps need to occur to allow the RNA-dependent polymerase to transition from an apo to a catalytically active state (Fig. 5c). The first takes place when the polymerase binds the template RNA. To facilitate RNA binding, the template channel has to undergo structural rearrangements. The thumb domain of the HIV-1 RT, for instance, has to move 20° relative to its position in the apo structure [132], whereas the HCV RdRp needs to contract its template channel from a width of 20.5 to 12.2 Å to interact with ssRNA [133]. In strong contrast, primer-dependent RdRps need to expand their template channel from a constricted apo form to one that can accommodate dsRNA [20, 27, 134]. In the PV RdRp this means a change from 15.7 to 18.7 Å [113]. Present evidence also suggests that the inherently flexible template channel of viral RNA-dependent polymerases can sample its conformational space rapidly [27, 133, 135], which thus makes it a very dynamic structure in apo form and able to respond quickly to an encounter with RNA.

The open complex that is formed after template binding is highly selective for base-pairing [20], in part due to motif D (see Sect. “The RNA-dependent polymerase domain”). In +RNA virus RdRps, correct nucleotide binding triggers the alignment of motif A to motif C and forces the N-terminal aspartate of motif A to bind metal ions [20]. This ostensibly subtle change completes the RRM-fold of the palm subdomain (the closed complex, Fig. 5a) and optimally positions the NTP for ribose binding by motifs A and B [20]. Interestingly, in some RNA polymerases the formation of the closed complex is fundamentally different. The HIV-1 RT, for instance, relies on large finger domain movements to close the active site and position the nascent RNA into the catalytic site, similar to DdDps [20, 132]. In the RdRps of dsRNA viruses, the RRM fold is already fully structured in the apo form, which means that only minor rearrangements of the side-chains are required to close their active site [17, 18].

The rate at which the residues in the active site sample their space is faster (*k* = 500−800 s^−1^) than the rate-limiting phosphoryl transfer step (*k* = 300 s^−1^) [136] (Fig. 5c, *k*_slowest_). Biochemical evidence suggests that the rate with which motif D takes the polymerase-RNA-NTP complex to a closed state is *k* = 520 s^−1^ [137], which is only somewhat faster than chemistry (Fig. 5c, *k*_slow_). So far the conformational change of motif D has only been observed with NMR [44], because it has proved too transient for crystallography. It is likely that the pre-catalytic conformational step is also dependent on the stabilisation of motifs A and F [27, 137].

After catalysis, the polymerase reverts to an open complex to allow translocation of the template-nascent strand duplex and pyrophosphate (PP_i_) release at *k* = 8,100 s^−1^ (Fig. 5c) [20, 136]. Viral polymerases that initiate de novo, however, must also transition from an initiation to an elongation conformation. As discussed in Sect. “Template recognition, initiation, elongation and regulation”, this involves rearrangements of several ångström in the thumb domain and the C-terminus of the ϕ6 RdRp [18] (Fig. 9b). Analogously, elements that stabilise the initiation of +RNA viruses such as the priming loop need to undergo a conformational change as well. Furthermore, such RdRps need to expand their template-nascent RNA exit channel again to facilitate the translocation of the now partially dsRNA template [133]. It is possible that such events, which only take place once during the synthesis of an RNA molecule, direct the polymerase briefly into a separate state or pause state (Fig. 5c), possibly akin to the pause states that can be observed during elongation [138]. Currently no experiments, which would likely require single-molecule techniques, have been performed to explore this.

### Fidelity

As discussed in Sect. “The RNA-dependent polymerase domain”, polymerase fidelity is largely determined by the conserved motifs of the polymerase domain. Furthermore, in vitro experiments have shown that the incorporation of nucleotides is indeed relatively robust [139, 140]. However, the error rate of RNA virus polymerases is nevertheless several orders of magnitude higher than the mutation rate of DNA genomes [141–143]. Moreover, it is also easily influenced by many environmental factors. The fidelity of the HIV-1 RT, for instance, can decrease 9-fold when the pH of the in vitro reaction is raised from pH 6.5 to pH 8.0 [144]. Similarly, nucleotide analogues can have a dramatic impact on the mutation rate of RNA virus RNA synthesis as well [145], as do various divalent metals [136].

It has been argued that the relative low fidelity of RNA virus polymerases is responsible for the ability of RNA viruses to overcome bottlenecks imposed by host defences and antivirals. Indeed, RNA virus strains that contained high-fidelity polymerase were attenuated in vivo [46, 146]. Moreover, the growth of IAV, PV, FMDV, *Chikungunya virus* (CHIKV), and *Human enterovirus 71* (EV71) in the presence of the nucleotide analogues such as ribavirin or 5-fluorouracil readily leads to the appearance resistance after several passages [147–151]. It is likely that the resistance mutations alter the positioning and dynamics of the motifs involved in nucleotide selection, which in turn optimises the RdRp’s ability to discriminate between NTPs [27].

In spite of the typically high error rate of viral RNA synthesis, dramatic mutations or deletions can be repaired. It was shown, for instance, that the RdRp of *Turnip crinkle virus* (TCV) is able to repair mutations in the 5′-CCUGCCC^OH^ sequence of the 3′-end of its genome using a 3-step process. Counter intuitively, the first step in this process is the non-templated synthesis of an RNA primer, which can next be aligned to the 3′-end and extended in a primer-dependent fashion [152]. A variant of this repair mechanism has been observed for the DV RdRp, provided that deletions were smaller than 6 nt and did not impair circularisation of the genome [153]. Although the use of non-templated nucleotide condensation for repair seems paradoxical, since the reaction mostly creates a pool of quasispecies, it is possible that the wild-type sequence is eventually ‘filtered’ back from the quasispecies population [153]. As non-templated polymerisation has been observed for −RNA, dsRNA, and +RNA RdRps [154–157]—in some cases for polyA tailing of viral transcripts [155]—it will be interesting to see if the conserved mechanism is universally used for template repair.

### Excision reaction

Although RNA virus polymerases do not contain a separate 3′-to-5′ exonuclease activity, they are able to perform a reversal of the polymerase reaction (Fig. 5c) that is typically referred to as pyrophosphorolysis. Hence, using PP_i_ and the same active site residues as those involved in nucleotide condensation, 3′ nucleoside monophosphates can be removed from the nascent strand, including misincorporated nucleotides or nucleoside analogues such as lamivudine (3TC), zidovudine (AZT), and 3′-dNTPs [158–160]. Crucially, chain-terminated complexes are stable in the presence of heparin, suggesting that pyrophosphorolysis typically occurs *in cis* [160].

Some RNA virus polymerases can substitute PP_i_ for an NTP molecule (typically ATP) [159]. These triphosphates are accommodated by an additional nucleoside binding pocket, which orients the γ-phosphate of the NTP towards the active site to accept the leaving monophosphate group [159]. In contrast to normal pyrophosphorolysis, this reaction results in the formation of dinucleoside tetraphosphate molecule. Through mutation of the NTP binding pocket, the NTP-dependent mechanism of excision can evolve into a mechanism that confers resistance to nucleoside analogues [132, 159, 161].

## Unknowns and closing remarks

The RdRp ensures that the viral genetic code is replicated and transcribed, but only for some viruses it is understood how these two processes are separated. In infections with *Alphaviridae,* for instance, the switch between the two processes is largely conducted by the viral protease, since incompletely processed polyproteins only support replication [162]. In contrast, it is still unclear what dedicates the IAV RdRp to either replication or transcription [163]. Various co-factors have been discovered that bias the activity of the RdRp towards one or the other pathway, including other viral proteins [164] and small viral RNAs (svRNA) [165]. In *Nidoviridae*, the regulation of transcription and replication also has to account for the synthesis of both full-length and multiple subgenome-length mRNAs [166]. Again, additional viral proteins have been proposed to guide this process, such as nsp1 of EAV [167], but the molecular details are still unclear.

Ultimately, our knowledge of RNA virus replication should lead to the discovery and development of new RNA virus inhibitors. A promising new inhibitor that has already been shown to affect the replication of many RNA viruses, including IAV, PA, WNV, YFV, and MACV, is the nucleotide analogue Favipiravir (T-705) [168]. Specific virus-specific inhibitors have also been found based on new polymerase structures. Co-crystallisation of IAV PB1 and PB2, for instance, has let to the discovery of peptides that interfere with RdRp assembly [169, 170]. In addition, screening assays are rapidly becoming invaluable for drug discovery and have led, for instance, to the identification of compounds that act as in vitro inhibitors of the IAV endonuclease activity [171]. Research has also demonstrated that general polymerase principles exist that may inspire strategies for a rapid and standardised design of live, attenuated vaccine strains [44]. In part, this latter insight relies on polymerase dynamics, which is also pointing to new druggable sites elsewhere in the structure [27, 133, 172].

In conclusion, it is clear that the RNA replication and transcription strategies vary greatly among RNA viruses. But many of the biochemical and biophysical properties of the polymerase domain have been uncovered, allowing for the development of novel antiviral strategies. Fundamental research into RNA-dependent polymerases is thus proving to be invaluable, and will likely continue to be, in combating current and emerging RNA virus infections.
